# Crystal structure of bis­{2-[(*E*)-(4-meth­oxy­lbenz­yl)imino­meth­yl]phenolato-κ^2^ *N*,*O* ^1^}nickel(II)

**DOI:** 10.1107/S160053681401650X

**Published:** 2014-07-23

**Authors:** Hadariah Bahron, Amalina Mohd Tajuddin, Wan Nazihah Wan Ibrahim, Hoong-Kun Fun, Suchada Chantrapromma

**Affiliations:** a Faculty of Applied Sciences, Universiti Teknologi MARA, 40450 Shah Alam, Selangor, Malaysia; b DDH CoRe, Universiti Teknologi MARA, 40450 Shah Alam, Selangor, Malaysia; c X-ray Crystallography Unit, School of Physics, Universiti Sains Malaysia, 11800 USM, Penang, Malaysia; d Department of Pharmaceutical Chemistry, College of Pharmacy, King Saud University, PO Box 2457, Riyadh 11451, Saudi Arabia; e Department of Chemistry, Faculty of Science, Prince of Songkla University, Hat-Yai, Songkhla 90112, Thailand

**Keywords:** crystal structure, nickel(II) complex, NO donors, Schiff base

## Abstract

The asymmetric unit of the title compound, [Ni(C_15_H_14_NO_2_)_2_], comprises an Ni^II^ cation, lying on an inversion centre, and a Schiff base anion that acts as a bidentate ligand. The Ni^II^ cation is in a square-planar coordination environment binding to the imine N and phenolate O atoms of the two Schiff base ligands. The N- and O-donor atoms of the two ligands are mutually *trans*, with Ni—N and Ni—O bond lengths of 1.9191 (11) and 1.8407 (9) Å, respectively. The plane of the meth­oxy­benzene ring makes a dihedral angle of 84.92 (6)° with that of the phenolate ring. In the crystal, mol­ecules are linked into screw chains by weak C—H⋯O hydrogen bonds. Additional C—H⋯O hydrogen bonds, together with C—H⋯π contacts, arrange the mol­ecules into sheets parallel to the *ac* plane.

## Abstract 2

The Ni^II^ atom in the title compound shows a square-planar NiN_2_O_2_ coordination with the imine N and phenolate O atoms of the two Schiff base ligands. C—H⋯O and C—H⋯π interactions result in the formation of sheets of molecules parallel to the *ac* plane.

## Chemical context   

Schiff bases have often been used as chelating ligands in coordination chemistry as they readily form stable complexes with most transition metal ions (Kalita *et al.*, 2014 ▶; Mohamed *et al.*, 2010 ▶). Metal complexes of Schiff bases containing nitro­gen and other donor atoms have received attention because of their stability, biological activity (Islam *et al.*, 2014 ▶) and potential applications in other fields, such as catalysis (Mohd Tajuddin *et al.*, 2012 ▶).
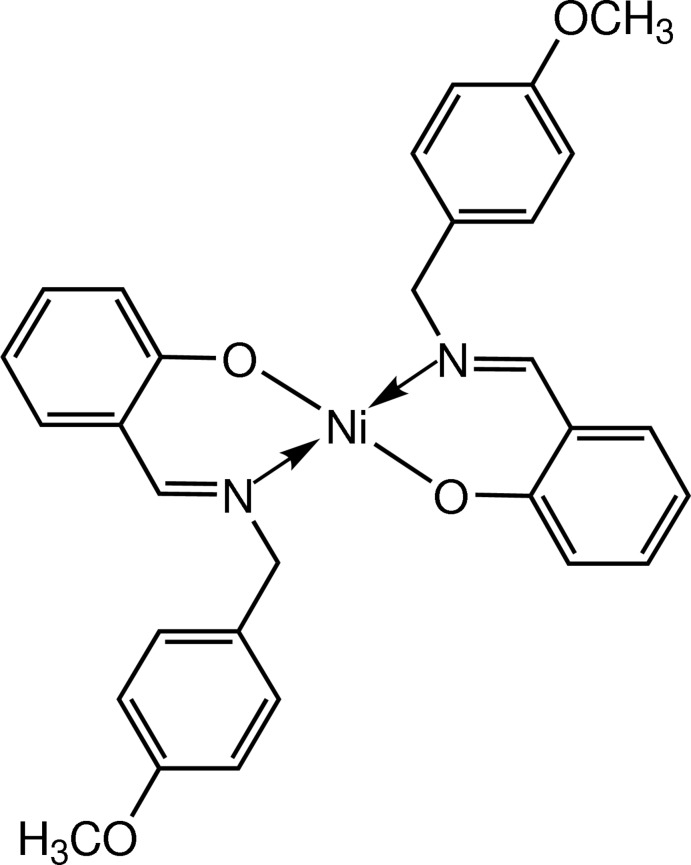


**Figure 1:**
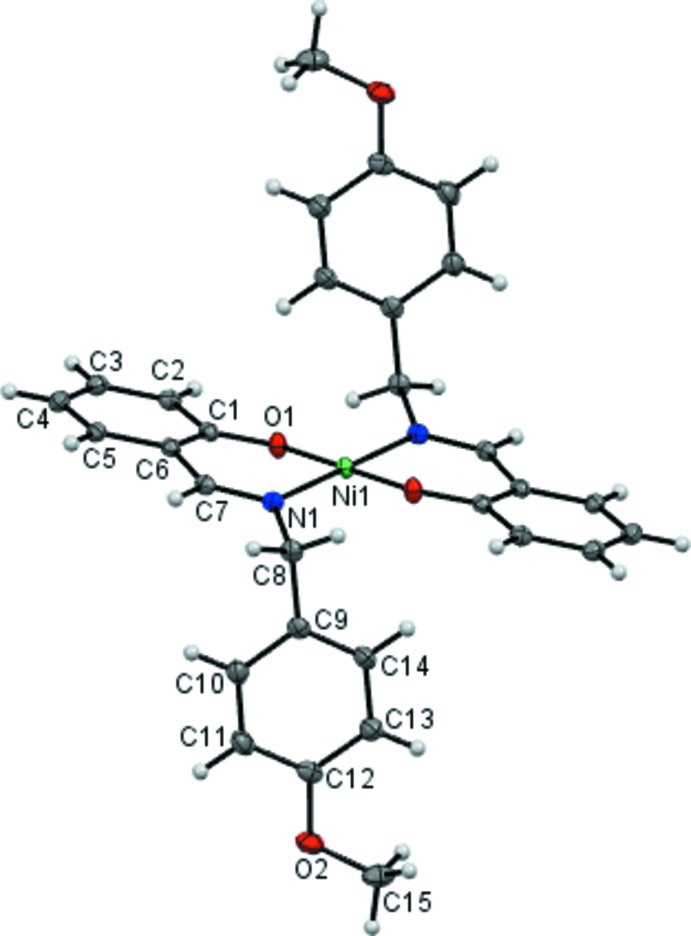
The mol­ecular structure of (I), showing 50% probability displacement ellipsoids and the atom-numbering scheme. The symmetry-related Schiff base ligand is generated by the symmetry code (−*x* + 1, −*y*, −*z* + 1).

The title compound, bis­{2-[(*E*)-(4-meth­oxy­lbenz­yl)imino­meth­yl]phenolato-κ^2^ *N*,*O* ^1^}nickel(II), (I), is related to bis­{2-[1-(benzyl­imino)­eth­yl]phenolato}palladium(II) (Mohd Tajuddin *et al.*, 2010 ▶) in terms of the geometry around the metal centre. However, we have extended our investigation to include a nickel compound with a Schiff base ligand that has a 4-meth­oxy substituent on the phenyl ring of the benzyl unit bound to the imine N atom (Fig. 1 ▶).

## Structural commentary   

The asymmetric unit of (I) consists of an Ni^II^ cation that lies on an inversion centre and a Schiff base anion that functions as a bidentate ligand (Fig. 1 ▶). The N_2_O_2_ donor set of the chelating Schiff base ligands has the N1 and O1 donor atoms mutually *trans*, in a distorted square-planar coordination geometry, with O1—Ni1—N1 = 92.30 (4)° and O1—Ni1—N1^i^ = 87.70 (4)° [symmetry code: (i) −*x* + 1, −*y*, −*z* + 1] and a maximum deviation from the NiN_2_O_2_ least-squares plane of 0.731 (1) Å for the N1 atom. The Ni1—N1 and Ni1—O1 bond lengths in the N_2_O_2_ coordination plane are 1.9191 (11) and 1.8407 (9) Å, respectively. These are similar to those observed in the other closely related Ni^II^ complexes with N_2_O_2_-coord­inating Schiff base ligands (Bahron *et al.*, 2011 ▶; Mohd Tajuddin *et al.*, 2010 ▶). Other bond lengths and angles observed in the structure are also normal. The meth­oxy substituent is coplanar with the ring to which it is bound, the C15—O2—C12—C13 torsion angle being 3.93 (2)°. The plane of the meth­oxy­benzene ring (C9–C14) makes a dihedral angle of 84.92 (6)° with that of the phenolate benzene ring (C1–C6). A weak intra­molecular C14—H14⋯O1 contact is also observed that affects the overall mol­ecular conformation.

## Supra­molecular features   

**Figure 2:**
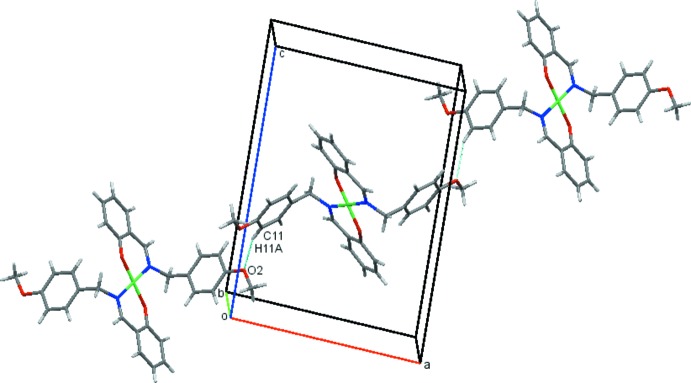
Screw chains of mol­ecules of (I) linked by C—H⋯O contacts (shown as dashed lines).

**Figure 3:**
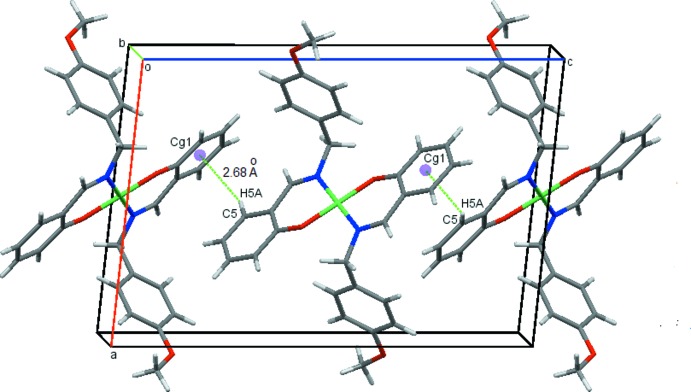
C—H⋯π contacts for (I), shown as dotted lines, with ring centroids shown as coloured spheres. *Cg*1 is the centroid of the C1–C6 ring.

**Figure 4:**
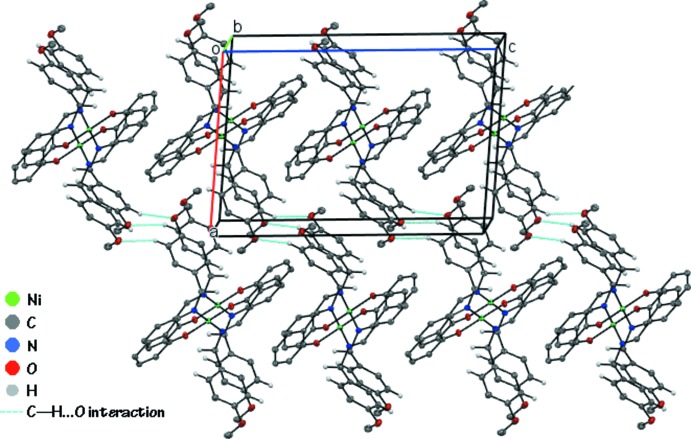
The packing of (I), viewed along the *b* axis, showing the stacking of sheets of Ni^II^ complex mol­ecules. Only H atoms involved in weak C—H⋯O inter­actions are shown for clarity.

In the crystal (Fig. 2 ▶), mol­ecules are linked into screw chains by weak C11—H11*A*⋯O2 inter­actions (Fig. 2 ▶ and Table 1 ▶). Additional C5—H5*A*··*·Cg*1 contacts link mol­ecules into chains along the *c-*axis direction (Fig. 3 ▶ and Table 1 ▶) resulting in sheets parallel to the *ac* plane and stacked along the *b* axis (Fig. 4 ▶).

## Database survey   

A search of the Cambridge Structural Database (Version 5.35, November 2013 with 3 updates; Allen, 2002 ▶) reveals a total of 1191 Ni^II^ complexes with an NiN_2_O_2_ coordination sphere. No fewer than 333 of these had the Ni^II^ atom chelated by two 3-(imino­meth­yl)phenolate residues. No corresponding structures with a benzyl or substituted benzyl unit bound to the imino N atom were found. However, extending the search to allow additional substitution on the phenolate ring resulted in seven discrete structures including the closely related bis­(2-[(*E*)-(4-fluoro­benz­yl)imino­meth­yl]-6-meth­oxy­phenolato-κ^2^ *N*,*O* ^1^)nickel(II) (Bahron *et al.*, 2011 ▶) and bis­{2-[(benzyl­imino)­meth­yl]-5-meth­oxy­phenolato}nickel(II) (Gou *et al.*, 2013 ▶)

## Synthesis and crystallization   

*N*-4-Meth­oxy­benzyl­salicyl­idene­imine (5 mmol, 0.6041 g) was dissolved in ethanol (15 ml). An ethano­lic solution of nickel(II) acetate tetra­hydrate (2.5 mmol, 0.6216 g) was added dropwise to the former solution and the mixture heated under reflux for 4 h, producing a green solid. The solid was filtered off, washed with ice-cold ethanol and air-dried at room temperature. The solid product was recrystallized from chloro­form, yielding green crystals (yield 43.3%; m.p. 469–472 K). Analytical data for [Ni(C_28_H_30_N_2_O_4_)]: C 66.82, H 5.23, N 5.19%; found: C 67.03, H 5.28, N 5.15%. IR (KBr, cm^−1^): ν(C=N) 1605 (*s*), ν(C—N) 1391 (*s*), ν(C—O) 1325 (*s*), ν(Ni—O) 598 (*w*), ν(Ni—N) 437 (*w*).

## Refinement   

Crystal data, data collection and structure refinement details are summarized in Table 2 ▶. All H atoms were positioned geometrically and allowed to ride on their parent atoms, with C—H = 0.95 for aromatic, 0.99 for CH_2_ and 0.98 Å for CH_3_ hydrogens. The *U*_iso_(H) values were constrained to be 1.5*U*_eq_ of the carrier atom for methyl H atoms and 1.2*U*_eq_ for the remaining H atoms. A rotating-group model was used for the methyl groups.

## Supplementary Material

Crystal structure: contains datablock(s) global, I. DOI: 10.1107/S160053681401650X/sj5419sup1.cif

Structure factors: contains datablock(s) I. DOI: 10.1107/S160053681401650X/sj5419Isup2.hkl

CCDC reference: 1014286

Additional supporting information:  crystallographic information; 3D view; checkCIF report

## Figures and Tables

**Table 1:** Hydrogen-bond geometry (Å, °) *Cg*1 is the centroid of the C1–C6 ring.

*D*—H⋯*A*	*D*—H	H⋯*A*	*D*⋯*A*	*D*—H⋯*A*
C11—H11*A*⋯O2^i^	0.95	2.47	3.3709 (17)	158
C14—H14*A*⋯O1^ii^	0.95	2.57	3.2281 (17)	126
C5—H5*A*⋯*Cg*1^iii^	0.95	2.68	3.3918 (13)	132

Symmetry codes: (i) 
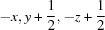
; (ii) 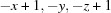; (iii) 
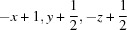
.

**Table 2:** Experimental details

Crystal data	
Chemical formula	[Ni(C_15_H_14_NO_2_)_2_]
*M*_r_	539.23
Crystal system, space group	Monoclinic, *P*2_1_/*c*
Temperature (K)	100
*a*, *b*, *c* (Å)	12.1847 (2), 5.6738 (1), 17.7620 (3)
β (°)	95.682 (1)
*V* (Å^3^)	1221.92 (4)
*Z*	2
Radiation type	Mo *K*α
μ (mm^−1^)	0.84
Crystal size (mm)	0.52 × 0.30 × 0.16

Data collection	
Diffractometer	Bruker APEXII CCD area detector
Absorption correction	Multi-scan (*SADABS*; Bruker, 2009 ▶)
*T*_min_, *T*_max_	0.670, 0.876
No. of measured, independent and observed [*I* > 2σ(*I*)] reflections	14541, 3542, 3092
*R*_int_	0.019
(sin θ/λ)_max_ (Å^−1^)	0.703

Refinement	
*R*[*F* ^2^ > 2σ(*F* ^2^)], *wR*(*F* ^2^), *S*	0.028, 0.074, 1.05
No. of reflections	3542
No. of parameters	170
H-atom treatment	H-atom parameters constrained
Δρ_max_, Δρ_min_ (e Å^−3^)	0.42, −0.32

Computer programs: *APEX2* and *SAINT* (Bruker, 2009 ▶), *SHELXTL* (Sheldrick, 2008 ▶), *PLATON* (Spek, 2009 ▶), *Mercury* (Macrae *et al.*, 2006 ▶) and *publCIF* (Westrip, 2010 ▶).

## supplementary crystallographic information

### Crystal data

**Table:**

[Ni(C_15_H_14_NO_2_)_2_]	*F*(000) = 564
*M**_r_* = 539.23	*D*_x_ = 1.466 Mg m^−^^3^
Monoclinic, *P*2_1_/*c*	Melting point = 469–472 K
Hall symbol: -P 2ybc	Mo *K*α radiation, λ = 0.71073 Å
*a* = 12.1847 (2) Å	Cell parameters from 3542 reflections
*b* = 5.6738 (1) Å	θ = 1.7–30.0°
*c* = 17.7620 (3) Å	µ = 0.84 mm^−^^1^
β = 95.682 (1)°	*T* = 100 K
*V* = 1221.92 (4) Å^3^	Needle, green
*Z* = 2	0.52 × 0.30 × 0.16 mm

### Data collection

**Table:**

Bruker APEXII CCD area-detector diffractometer	3542 independent reflections
Radiation source: sealed tube	3092 reflections with *I* > 2σ(*I*)
Graphite monochromator	*R*_int_ = 0.019
φ and ω scans	θ_max_ = 30.0°, θ_min_ = 1.7°
Absorption correction: multi-scan (*SADABS*; Bruker, 2009)	*h* = −17→17
*T*_min_ = 0.670, *T*_max_ = 0.876	*k* = −7→7
14541 measured reflections	*l* = −24→24

### Refinement

**Table:**

Refinement on *F*^2^	Primary atom site location: structure-invariant direct methods
Least-squares matrix: full	Secondary atom site location: difference Fourier map
*R*[*F*^2^ > 2σ(*F*^2^)] = 0.028	Hydrogen site location: inferred from neighbouring sites
*wR*(*F*^2^) = 0.074	H-atom parameters constrained
*S* = 1.05	*w* = 1/[σ^2^(*F*_o_^2^) + (0.0313*P*)^2^ + 0.7976*P*] where *P* = (*F*_o_^2^ + 2*F*_c_^2^)/3
3542 reflections	(Δ/σ)_max_ < 0.001
170 parameters	Δρ_max_ = 0.42 e Å^−^^3^
0 restraints	Δρ_min_ = −0.32 e Å^−^^3^

### Special details

**Table:**

Experimental. The crystal was placed in the cold stream of an Oxford Cryosystems Cobra open-flow nitrogen cryostat (Cosier & Glazer, 1986) operating at 100.0 (1) K.
Geometry. All e.s.d.'s (except the e.s.d. in the dihedral angle between two l.s. planes) are estimated using the full covariance matrix. The cell e.s.d.'s are taken into account individually in the estimation of e.s.d.'s in distances, angles and torsion angles; correlations between e.s.d.'s in cell parameters are only used when they are defined by crystal symmetry. An approximate (isotropic) treatment of cell e.s.d.'s is used for estimating e.s.d.'s involving l.s. planes.
Refinement. Refinement of *F*^2^ against ALL reflections. The weighted *R*-factor *wR* and goodness of fit *S* are based on *F*^2^, conventional *R*-factors *R* are based on *F*, with *F* set to zero for negative *F*^2^. The threshold expression of *F*^2^ > σ(*F*^2^) is used only for calculating *R*-factors(gt) *etc*. and is not relevant to the choice of reflections for refinement. *R*-factors based on *F*^2^ are statistically about twice as large as those based on *F*, and *R*- factors based on ALL data will be even larger.

### Fractional atomic coordinates and isotropic or equivalent isotropic displacement parameters (Å^2^)

**Table:**

	*x*	*y*	*z*	*U*_iso_*/*U*_eq_	
Ni1	0.5000	0.0000	0.5000	0.01253 (7)	
O1	0.58126 (8)	−0.03642 (16)	0.41876 (5)	0.01676 (19)	
O2	−0.03916 (8)	−0.2287 (2)	0.34237 (6)	0.0272 (2)	
N1	0.42000 (8)	0.2622 (2)	0.45360 (6)	0.0142 (2)	
C1	0.60209 (10)	0.1191 (2)	0.36758 (6)	0.0141 (2)	
C2	0.68624 (10)	0.0696 (3)	0.31981 (7)	0.0164 (2)	
H2A	0.7260	−0.0744	0.3255	0.020*	
C3	0.71053 (10)	0.2297 (3)	0.26525 (7)	0.0176 (2)	
H3A	0.7676	0.1945	0.2343	0.021*	
C4	0.65287 (11)	0.4428 (3)	0.25451 (7)	0.0182 (3)	
H4A	0.6698	0.5501	0.2163	0.022*	
C5	0.57101 (10)	0.4940 (2)	0.30048 (7)	0.0157 (2)	
H5A	0.5315	0.6380	0.2938	0.019*	
C6	0.54531 (10)	0.3354 (2)	0.35715 (6)	0.0138 (2)	
C7	0.45329 (10)	0.3875 (2)	0.39913 (7)	0.0146 (2)	
H7A	0.4130	0.5271	0.3857	0.018*	
C8	0.31199 (10)	0.3363 (2)	0.47834 (7)	0.0163 (2)	
H8A	0.2966	0.5015	0.4629	0.020*	
H8B	0.3151	0.3279	0.5342	0.020*	
C9	0.22048 (10)	0.1787 (2)	0.44360 (7)	0.0159 (2)	
C10	0.16960 (11)	0.2254 (3)	0.37107 (7)	0.0209 (3)	
H10A	0.1939	0.3559	0.3436	0.025*	
C11	0.08462 (11)	0.0860 (3)	0.33834 (7)	0.0235 (3)	
H11A	0.0517	0.1204	0.2888	0.028*	
C12	0.04753 (10)	−0.1046 (3)	0.37812 (7)	0.0194 (3)	
C13	0.09757 (11)	−0.1573 (3)	0.44988 (8)	0.0216 (3)	
H13A	0.0735	−0.2887	0.4770	0.026*	
C14	0.18353 (11)	−0.0150 (3)	0.48172 (8)	0.0203 (3)	
H14A	0.2176	−0.0518	0.5307	0.024*	
C15	−0.08444 (12)	−0.4144 (3)	0.38380 (9)	0.0274 (3)	
H15A	−0.1488	−0.4812	0.3537	0.041*	
H15B	−0.1069	−0.3521	0.4314	0.041*	
H15C	−0.0287	−0.5376	0.3947	0.041*	

### Atomic displacement parameters (Å^2^)

**Table:**

	*U*^11^	*U*^22^	*U*^33^	*U*^12^	*U*^13^	*U*^23^
Ni1	0.01363 (11)	0.01119 (12)	0.01281 (10)	0.00205 (8)	0.00150 (7)	0.00034 (8)
O1	0.0207 (4)	0.0148 (5)	0.0153 (4)	0.0041 (4)	0.0044 (3)	0.0021 (3)
O2	0.0239 (5)	0.0353 (6)	0.0210 (5)	−0.0062 (5)	−0.0041 (4)	−0.0053 (4)
N1	0.0138 (4)	0.0131 (5)	0.0157 (4)	0.0010 (4)	0.0010 (3)	−0.0012 (4)
C1	0.0146 (5)	0.0148 (6)	0.0126 (5)	−0.0009 (5)	−0.0009 (4)	−0.0015 (4)
C2	0.0158 (5)	0.0168 (6)	0.0165 (5)	0.0003 (5)	0.0013 (4)	−0.0021 (5)
C3	0.0159 (5)	0.0198 (7)	0.0174 (5)	−0.0029 (5)	0.0025 (4)	−0.0026 (5)
C4	0.0187 (5)	0.0181 (6)	0.0179 (5)	−0.0039 (5)	0.0016 (4)	0.0015 (5)
C5	0.0156 (5)	0.0133 (6)	0.0176 (5)	−0.0021 (5)	−0.0007 (4)	0.0011 (5)
C6	0.0144 (5)	0.0134 (6)	0.0133 (5)	−0.0008 (4)	−0.0009 (4)	−0.0009 (4)
C7	0.0149 (5)	0.0123 (6)	0.0160 (5)	0.0010 (5)	−0.0012 (4)	−0.0009 (5)
C8	0.0159 (5)	0.0149 (6)	0.0183 (5)	0.0039 (5)	0.0029 (4)	−0.0004 (5)
C9	0.0140 (5)	0.0171 (6)	0.0167 (5)	0.0049 (5)	0.0026 (4)	−0.0008 (5)
C10	0.0201 (6)	0.0250 (7)	0.0178 (5)	0.0023 (5)	0.0027 (4)	0.0036 (5)
C11	0.0221 (6)	0.0340 (8)	0.0141 (5)	0.0021 (6)	0.0000 (4)	0.0007 (6)
C12	0.0163 (5)	0.0243 (7)	0.0174 (5)	0.0023 (5)	0.0006 (4)	−0.0057 (5)
C13	0.0212 (6)	0.0214 (7)	0.0216 (6)	−0.0012 (5)	−0.0007 (5)	0.0019 (5)
C14	0.0187 (6)	0.0227 (7)	0.0185 (6)	0.0009 (5)	−0.0030 (4)	0.0029 (5)
C15	0.0219 (6)	0.0282 (8)	0.0317 (7)	−0.0039 (6)	0.0007 (5)	−0.0100 (7)

### Geometric parameters (Å, º)

**Table:**

Ni1—O1	1.8407 (9)	C6—C7	1.4374 (16)
Ni1—O1^i^	1.8407 (9)	C7—H7A	0.9500
Ni1—N1	1.9191 (11)	C8—C9	1.5123 (18)
Ni1—N1^i^	1.9191 (11)	C8—H8A	0.9900
O1—C1	1.3095 (15)	C8—H8B	0.9900
O2—C12	1.3717 (16)	C9—C14	1.3892 (19)
O2—C15	1.427 (2)	C9—C10	1.3986 (17)
N1—C7	1.2977 (16)	C10—C11	1.384 (2)
N1—C8	1.4887 (15)	C10—H10A	0.9500
C1—C6	1.4123 (18)	C11—C12	1.392 (2)
C1—C2	1.4221 (16)	C11—H11A	0.9500
C2—C3	1.3813 (18)	C12—C13	1.3898 (18)
C2—H2A	0.9500	C13—C14	1.3967 (19)
C3—C4	1.402 (2)	C13—H13A	0.9500
C3—H3A	0.9500	C14—H14A	0.9500
C4—C5	1.3810 (17)	C15—H15A	0.9800
C4—H4A	0.9500	C15—H15B	0.9800
C5—C6	1.4080 (17)	C15—H15C	0.9800
C5—H5A	0.9500		
			
O1—Ni1—O1^i^	180.000 (1)	C6—C7—H7A	116.9
O1—Ni1—N1	92.30 (4)	N1—C8—C9	110.50 (10)
O1^i^—Ni1—N1	87.70 (4)	N1—C8—H8A	109.5
O1—Ni1—N1^i^	87.70 (4)	C9—C8—H8A	109.5
O1^i^—Ni1—N1^i^	92.30 (4)	N1—C8—H8B	109.5
N1—Ni1—N1^i^	180.00 (6)	C9—C8—H8B	109.5
C1—O1—Ni1	128.67 (8)	H8A—C8—H8B	108.1
C12—O2—C15	117.36 (11)	C14—C9—C10	117.60 (12)
C7—N1—C8	114.59 (11)	C14—C9—C8	122.03 (11)
C7—N1—Ni1	124.19 (9)	C10—C9—C8	120.37 (12)
C8—N1—Ni1	121.22 (8)	C11—C10—C9	121.56 (13)
O1—C1—C6	123.41 (11)	C11—C10—H10A	119.2
O1—C1—C2	118.82 (12)	C9—C10—H10A	119.2
C6—C1—C2	117.77 (11)	C10—C11—C12	119.86 (12)
C3—C2—C1	120.40 (12)	C10—C11—H11A	120.1
C3—C2—H2A	119.8	C12—C11—H11A	120.1
C1—C2—H2A	119.8	O2—C12—C13	124.20 (13)
C2—C3—C4	121.55 (12)	O2—C12—C11	115.95 (12)
C2—C3—H3A	119.2	C13—C12—C11	119.84 (13)
C4—C3—H3A	119.2	C12—C13—C14	119.37 (13)
C5—C4—C3	118.84 (12)	C12—C13—H13A	120.3
C5—C4—H4A	120.6	C14—C13—H13A	120.3
C3—C4—H4A	120.6	C9—C14—C13	121.74 (12)
C4—C5—C6	120.87 (12)	C9—C14—H14A	119.1
C4—C5—H5A	119.6	C13—C14—H14A	119.1
C6—C5—H5A	119.6	O2—C15—H15A	109.5
C5—C6—C1	120.55 (11)	O2—C15—H15B	109.5
C5—C6—C7	118.67 (12)	H15A—C15—H15B	109.5
C1—C6—C7	120.51 (11)	O2—C15—H15C	109.5
N1—C7—C6	126.28 (12)	H15A—C15—H15C	109.5
N1—C7—H7A	116.9	H15B—C15—H15C	109.5
			
N1—Ni1—O1—C1	22.59 (11)	Ni1—N1—C7—C6	9.30 (18)
N1^i^—Ni1—O1—C1	−157.41 (11)	C5—C6—C7—N1	−178.90 (12)
O1—Ni1—N1—C7	−19.81 (11)	C1—C6—C7—N1	7.05 (19)
O1—Ni1—N1—C8	159.85 (9)	C7—N1—C8—C9	99.88 (13)
O1^i^—Ni1—N1—C8	−20.15 (9)	Ni1—N1—C8—C9	−79.80 (11)
Ni1—O1—C1—C6	−13.67 (17)	N1—C8—C9—C14	95.30 (14)
Ni1—O1—C1—C2	166.19 (9)	N1—C8—C9—C10	−84.93 (14)
O1—C1—C2—C3	−179.94 (11)	C14—C9—C10—C11	0.7 (2)
C6—C1—C2—C3	−0.06 (18)	C8—C9—C10—C11	−179.09 (12)
C1—C2—C3—C4	−0.79 (19)	C9—C10—C11—C12	0.5 (2)
C2—C3—C4—C5	0.89 (19)	C15—O2—C12—C13	3.9 (2)
C3—C4—C5—C6	−0.13 (19)	C15—O2—C12—C11	−175.51 (13)
C4—C5—C6—C1	−0.71 (18)	C10—C11—C12—O2	178.05 (12)
C4—C5—C6—C7	−174.76 (11)	C10—C11—C12—C13	−1.4 (2)
O1—C1—C6—C5	−179.33 (11)	O2—C12—C13—C14	−178.31 (13)
C2—C1—C6—C5	0.80 (17)	C11—C12—C13—C14	1.1 (2)
O1—C1—C6—C7	−5.40 (18)	C10—C9—C14—C13	−1.0 (2)
C2—C1—C6—C7	174.74 (11)	C8—C9—C14—C13	178.78 (12)
C8—N1—C7—C6	−170.38 (11)	C12—C13—C14—C9	0.1 (2)

Symmetry code: (i) −*x*+1, −*y*, −*z*+1.

### Hydrogen-bond geometry (Å, º)

*Cg*1 is the centroid of the C1–C6 ring.

**Table:**

*D*—H···*A*	*D*—H	H···*A*	*D*···*A*	*D*—H···*A*
C11—H11*A*···O2^ii^	0.95	2.47	3.3709 (17)	158
C14—H14*A*···O1^i^	0.95	2.57	3.2281 (17)	126
C5—H5*A*···*Cg*1^iii^	0.95	2.68	3.3918 (13)	132

Symmetry codes: (i) −*x*+1, −*y*, −*z*+1; (ii) −*x*, *y*+1/2, −*z*+1/2; (iii) −*x*+1, *y*+1/2, −*z*+1/2.
